# Microbiological Study in Petrol-Spiked Soil

**DOI:** 10.3390/molecules26092664

**Published:** 2021-05-01

**Authors:** Agata Borowik, Jadwiga Wyszkowska, Jan Kucharski

**Affiliations:** Department of Soil Science and Microbiology, University of Warmia and Mazury in Olsztyn, 10-727 Olsztyn, Poland; agata.borowik@uwm.edu.pl (A.B.); jan.kucharski@uwm.edu.pl (J.K.)

**Keywords:** petrol, microbial diversity, soil, degradation hydrocarbons, phytoremediation, soil enzymes

## Abstract

The pollution of arable lands and water with petroleum-derived products is still a valid problem, mainly due the extensive works aimed to improve their production technology to reduce fuel consumption and protect engines. An example of the upgraded fuels is the BP 98 unleaded petrol with Active technology. A pot experiment was carried out in which Eutric Cambisol soil was polluted with petrol to determine its effect on the microbiological and biochemical properties of this soil. Analyses were carried out to determine soil microbiome composition—with the incubation and metagenomic methods, the activity of seven enzymes, and cocksfoot effect on hydrocarbon degradation. The following indices were determined: colony development index (CD); ecophysiological diversity index (EP); index of cocksfoot effect on soil microorganisms and enzymes (IF_G_); index of petrol effect on soil microorganisms and enzymes (IF_P_); index of the resistance of microorganisms, enzymes, and cocksfoot to soil pollution with petrol (RS); Shannon–Weaver’s index of bacterial taxa diversity (H); and Shannon–Weaver’s index of hydrocarbon degradation (ID_H_). The soil pollution with petrol was found to increase population numbers of bacteria and fungi, and *Protebacteria* phylum abundance as well as to decrease the abundance of *Actinobacteria* and *Acidobacteria* phyla. The cultivation of cocksfoot on the petrol-polluted soil had an especially beneficial effect mainly on the bacteria belonging to the *Ramlibacter*, *Pseudoxanthomonas*, *Mycoplana*, and *Sphingobium* genera. The least susceptible to the soil pollution with petrol and cocksfoot cultivation were the bacteria of the following genera: *Kaistobacter*, *Rhodoplanes*, *Bacillus*, *Streptomyces*, *Paenibacillus*, *Phenylobacterium*, and *Terracoccus*. Cocksfoot proved effective in the phytoremediation of petrol-polluted soil, as it accelerated hydrocarbon degradation and increased the genetic diversity of bacteria. It additionally enhanced the activities of soil enzymes.

## 1. Introduction

The following four elements are the main determinants of human and animal health: soil fertility, water quality, air purity, and technologies employed in the agri-food processing [1,2,3]. Each of these elements is crucial to the sustainable development of populations, with soil playing a significant role in this chain due to its quality being the main driver of the dietary value of agricultural crops produced [4,5]. The physical and chemical devastation of soil, including both the area-related one affected by the widely understood industrialization, and the point-related one associated with incidental events, urges for the continuous upgrade of various reclamation technologies [6,7,8]. An increasingly important role is ascribed in this regard to microorganisms, as evidenced by the bioaugmentation-aided reclamation methods [9,10,11,12].

The soil microbiome is highly diversified [13,14]. According to Nesme [15], one gram of soil can provide a habitat for over 10,000 various bacterial species. However, the assessment of soil ecosystem functioning is usually based on the physical soil indicators related to its texture, aggregation, porosity, and humidity [16,17,18,19], as well as the chemical ones, including the contents of organic carbon [20,21], total nitrogen [22], available phosphorus and potassium [23,24], and also soil pH value [25,26]. The methods used for soil quality assessment usually take no account of the microbiological processes ongoing in the soil, such as e.g., ISO standards [27]. Furthermore, Bünemann et al. [28] have underlined the underestimation of biological indicators in the soil functioning evaluation. It is an extremely valuable observation considering the meaningful role the soil microorganisms play in the structure and functioning of ecosystems [29]. The life on Earth would not be possible without them [28,30]. The communities of rhizospheric microorganisms are involved in, i.e., soil structure formation [18] and organic matter degradation [31,32], as well as increase nutrient availability and plant productivity [33,34]. Root secretions are both perfect substrates and signalling molecules for microorganisms, establishing complex interactions between roots, soil, and microorganisms [35,36,37]. This complex community of microorganisms associated with plants is also called the second plant genome. Plants can influence the development of their rhizospheric microbiome [38]. Hence, the soil microorganisms are of key importance to plant health and to the biogeochemical cycles of biogenic elements [20,22,32,39,40].

Both the soil microorganisms and the enzymes they produce are sensitive to varying environmental conditions [41,42,43,44]. They faithfully reproduce the condition of the soil environment; therefore, the microbiological and biochemical indicators cannot be neglected in soil quality evaluation [6,13,30,45].

The rapid development of molecular methods observed in the last decade has caused the appearance of an increasing number of indicators based on genotypic and phenotypic diversity, next to the microbiological ones determined with conventional methods [46,47]. They allow for the immediate assessment of the composition and functioning of a microbial community at various trophic levels [14,48]. Thus, the molecular methods complement the conventional microbiological and biochemical indicators of soil quality [49]. Nevertheless, the molecular techniques are also burdened with some errors resulting from sample contamination, primer selection, or taxonomic classification techniques [15,50]. Moreover, a large proportion of soil organisms has not yet been characterized in terms of their taxonomy and functions [30,46]. Therefore, there has long been a debate about the best choice of bioindicators that can objectively be used to monitor soil quality and thus provide early warning of a potential loss of its multifunctionality [30]. It is important to apply a holistic approach to soil quality assessment, taking into account different ecosystems. However, this assessment may be distorted by the specific, often contradictory, response of microorganisms to heavy metals [51,52] and organic pollutants [6,12,53,54,55].

Many functions performed by soil microorganisms are currently under threat due to the degradation of soil ecosystems by petroleum-derived products, which is a global problem [56]. The stability of microorganisms is disturbed in such an environment [41,57]. Soil pollution with petroleum-derived products induces stress in microbial communities that require long adaptation periods [43,58]. According to Borowik et al. [59], diesel oil triggered greater changes in the soil microbiome than unleaded petrol. Soil pollution with diesel oil reduced the counts of all bacterial taxa except for species, while soil pollution with petrol decreased the bacterial diversity only at the class, order, and family level. The composition of soil microbiota in the soil polluted with petrol hydrocarbons evolves [53,60]. The soils exposed to the pressure of PAHs are mainly predominated by representatives of *β*-, *γ*-*Proteobacteria*, *Actinobacteria*, and *Bacteroidia* [61,62,63]. As Tejeda-Agredano et al. [64] claim, the prevailing genera of the soils polluted with petroleum-derived products include *Sphingomonas*, *Commamonas*, *Oxalobacteria*, and *Xhanthomonas*, whereas according to Kumar et al. [65] these are the representatives of *Alcanivorax* and *Aequorivita*. Soil pollution with petroleum-derived products not only causes changes in the microbial diversity but also, through the food chain, contributes to the induction of chronic diseases of immunosuppressed men and animals. These products pose a significant threat to the environment and ecological safety of the global population [4], thereby reducing the agricultural productivity of soils [5]. Therefore, restoring the soil biological homeostasis is a priority to maintain the social stability and sustainable development [66,67], and to ensure food safety [3,53]. This restoration will be feasible owing to the more comprehensive understanding of the interactions of soil microorganisms under various stress conditions, which will additionally enable predicting responses of the soil microbial communities and activities of enzymes in the environment exposed to the pressure of petroleum-based products. The above premises have prompted a research aimed to determine the impact of soil pollution with petrol on its microbiome and enzymatic activity, on the response of cocksfoot to the soil pollution, and the role of this plant in degradation of petrol hydrocarbons.

## 2. Results

### 2.1. Microbiological Properties of Soil

**Figure 1:**
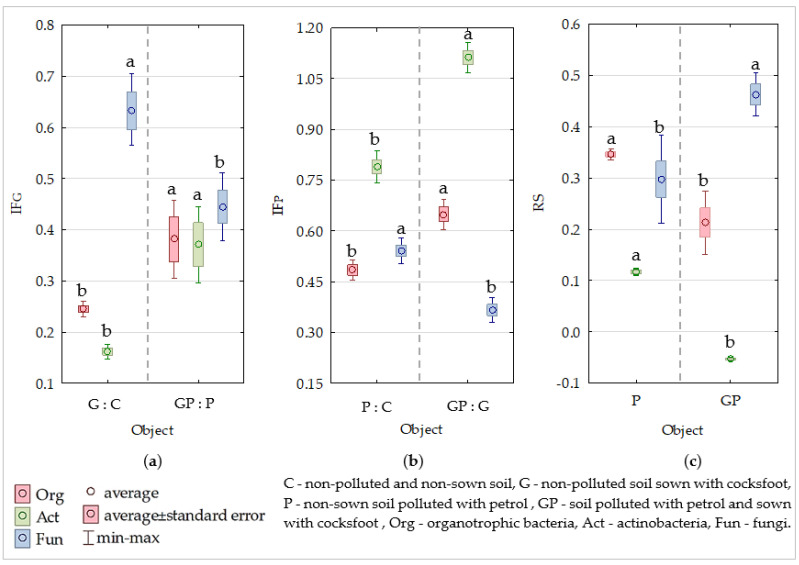
Indices effect: (**a**) of cocksfoot on soil microorganisms (IF_G_), (**b**) of petrol on soil microorganisms (IF_P_), and (**c**) of resistance (RS) of soil microorganisms to the effects of petrol (P). Homogeneous groups denoted with letters (a,b) were calculated separately for each of the microorganisms.

Cocksfoot was found to exert a beneficial effect on organotrophic bacteria (Org), actinobacteria (Act), and fungi (Fun), as indicated by the positive values of the IF_G_ index, ranging from 0.162 (Act) to 0.629 (Table S1, Figure 1a). Intermediate values of IF_G_ were determined for Org. The pollution of non-sown soil and soil sown with cocksfoot with petrol significantly promoted the proliferation of all tested groups of microorganisms, as evidenced by positive IF_P_ values. Petrol had a stronger effect on the organotrophic bacteria and actinobacteria in the soil planted with cocksfoot, whereas its impact on fungi in this soil was weaker. These effects were reflected in the values of the index of microorganism resistance (RS) to petrol (Figure 1a–c).

**Figure 2:**
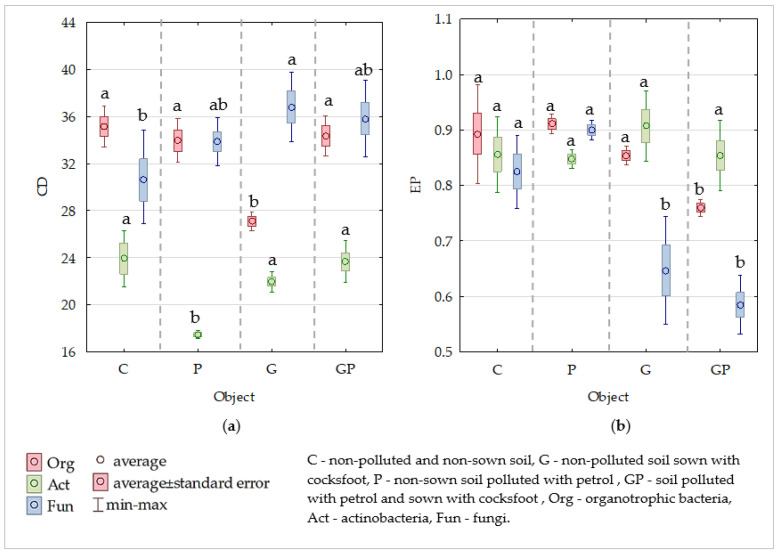
Effect of petrol (P) and cocksfoot (G) on changes in (**a**) the colony development (CD) index, and (**b**) soil microorganisms and the value of their ecophysiological diversity (EP) index. Homogeneous groups denoted with letters (a,b) were calculated separately for each of the microorganisms.

Cocksfoot cultivation in the soil tested contributed to a decrease in the colony development (CD) index of organotrophic bacteria and actinobacteria, and to an increase in the CD index of fungi. The CD index values were similarly affected by soil pollution with petrol. In the non-sown soil, petrol caused an increase in the ecophysiological diversity (EP) index of organotrophic bacteria and fungi, whereas in the soil sown with cocksfoot, it decreased EP index values of all microorganisms tested (Figure 2a,b).

**Figure 3:**
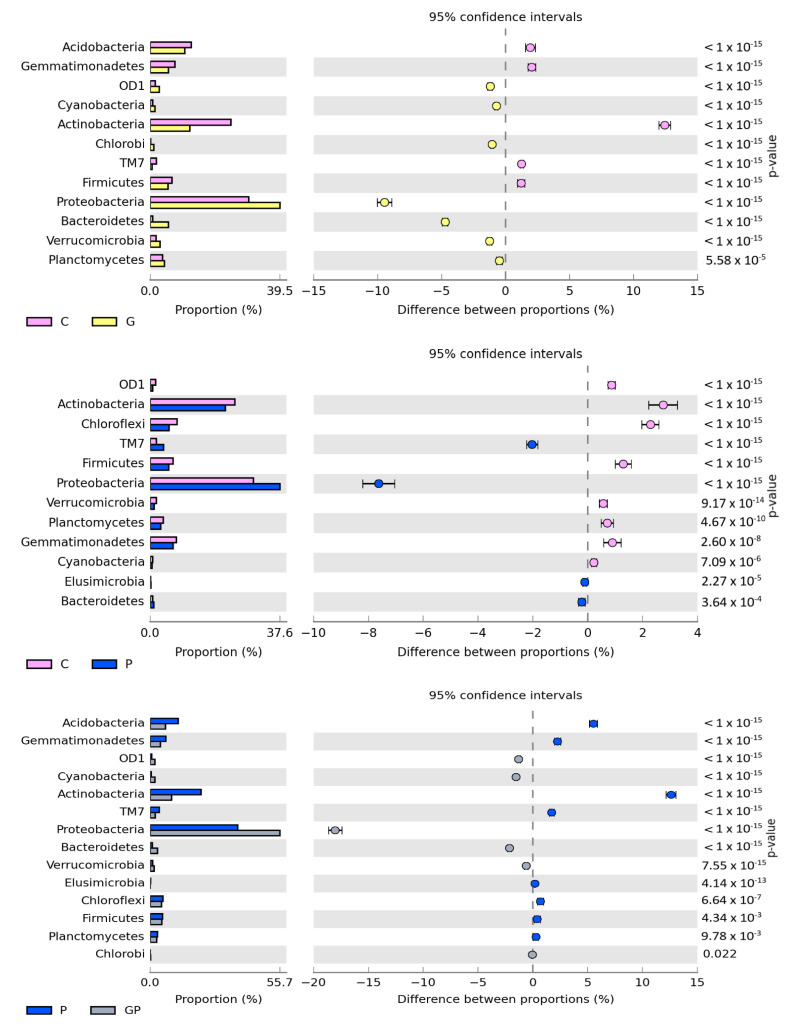

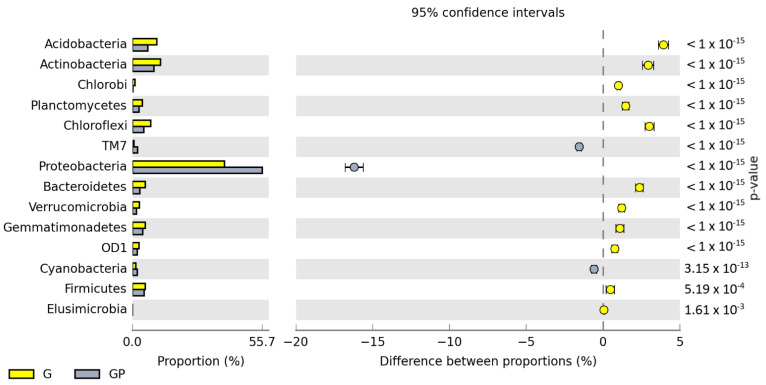
Comparison of the relative abundance of bacterial phyla in the soil between particular pots, with difference between the proportions at ≥1%. C—non-polluted and non-sown soil, G—non-polluted soil sown with cocksfoot, P—non-sown soil polluted with petrol, and GP—soil polluted with petrol and sown with cocksfoot.

The number of isolated OTUs reached 56,726 in the non-sown and non-polluted soil and 49,640 in the non-sown and petrol-polluted soil. After cocksfoot sowing, OTU numbers reached 59,104 and 54,338 in the non-polluted and polluted soil. Regardless of soil cultivation type and pollution with petrol, the prevailing phyla were: *Proteobacteria*, *Actinobacteria*, and *Acidobacteria* (Figure 3). The pollution of non-sown soil increased the OTU number of *Proteobacteria* by 7.67% and that of *TM7* by 2.03%, but decreased OTU numbers of *Actinobacteria* and *Chloroflexi* by 2.73% and 2.28%, respectively. In turn, cocksfoot sowing increased the OUT number of *Proteobacteria* by 16.18% and decreased the OUT number of *Acidobacteria* by 3.95%, that of *Chloroflexi* by 3.02%, that of *Actinobacteria* by 2.94%, and that of *Bacteroidetes* by 2.37%, compared to the soil sown with cocksfoot but not polluted with petrol. The greatest differences in OTU numbers of the prevailing phyla were found when comparing the non-sown petrol-polluted soil with the soil sown with cocksfoot but not polluted with petrol (P vs. GP). Cocksfoot sowing in the petrol-polluted soil increased OTU numbers of *Proteobacteria* by 17.97% and *Bacteroidetes* by 2.14%, whereas it decreased those of *Actinobacteria* by 12.66%, *Acidobacteria* by 5.54%, and *Gemmatimonadetes* by 2.27%.

**Figure 4:**
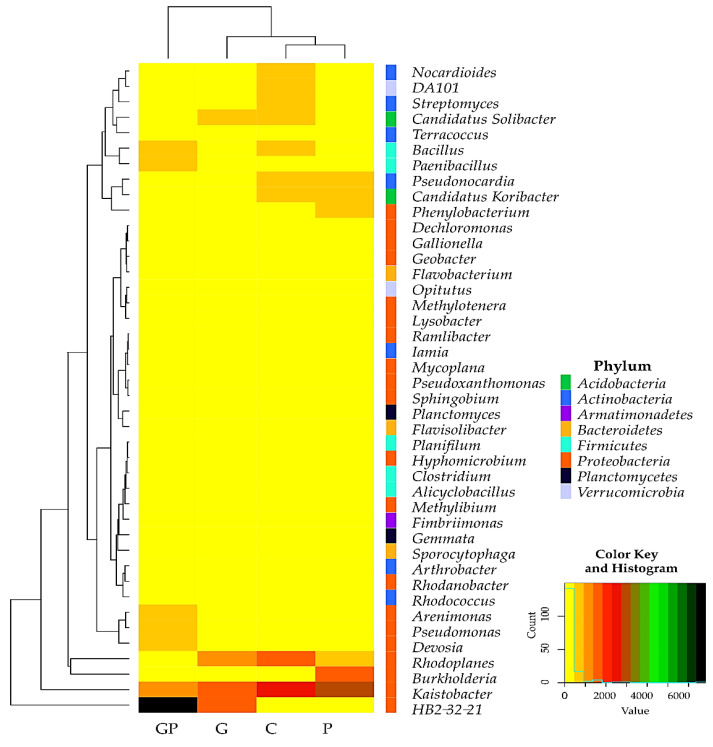
Number of OTU ≥ 1% of 42 bacterial genera in the soil presented using the heat map with classification to the phylum. C—non-polluted and non-sown soil, G—non-polluted soil sown with cocksfoot, P—non-sown soil polluted with petrol, and GP—soil polluted with petrol and sown with cocksfoot.

The bacterial structure was observed to change successively depending on soil cultivation and pollution with petrol. In total, 42 genera were identified in the soil samples that were represented by at least 1% of total assigned sequences (Figure 4). Twenty of them (*Arenimonas*, *Burkholderia*, *Dechloromonas*, *Devosia*, *Gallionella*, *Geobacter*, *HB2-32-21*, *Hyphomicrobium*, *Kaistobacter*, *Lysobacter*, *Methylibium*, *Methylotenera*, *Mycoplana*, *Phenylobacterium*, *Pseudomonas*, *Pseudoxanthomonas*, *Ramlibacter*, *Rhodanobacter*, *Rhodoplanes*, and *Sphingobium*) were classified to the phylum *Proteobacteria*, seven of them (*Arthrobacter*, *Iamia*, *Nocardioides*, *Pseudonocardia*, *Rhodococcus*, *Streptomyces*, and *Terracoccus*) to *Actinobacteria*, five (*Alicyclobacillus*, *Bacillus*, *Clostridium*, *Paenibacillus*, and *Planifilum*) to *Firmicutes*, three (*Flavobacterium*, *Flavisolibacter*, *and Sporocytophaga*) to *Bacteroidetes*, two (*DA101* and *Opitutus*) to *Verrucomicrobia*, two (*Candidatus Koribacter* and *Candidatus Solibacter*) to *Acidobacteria*, two (*Gemmata* and *Planctomyces*) to *Planctomycetes*, and one (*Fimbriimonas*) to *Armatimonadetes*. In the non-sown soil polluted and not polluted with petrol, the most abundant genus turned out to be *Kaistobacter*, which accounted for 17.55% and 21.74% of all assigned sequences, respectively. In turn, *HB2-32-21* turned out to be the most abundant genus in the soil sown with cocksfoot, accounting for 8.59% and for as much as 31.40% of total OTUs in the non-polluted and polluted soils, respectively. The structure of individual identified genera varied depending on soil cultivation type and its pollution with petrol. The pollution of non-sown soil was found to increase the relative abundance of *Kaistobacter*, *Burkholderia*, and *Phenylobacterium*, whereas the pollution of soil sown with cocksfoot—to increase the relative abundance of *HB2-32-21, Devosia*, *Bacillus*, *Pseudomonas*, *Arenimonas*, and *Paenibacillus*.

**Figure 5:**
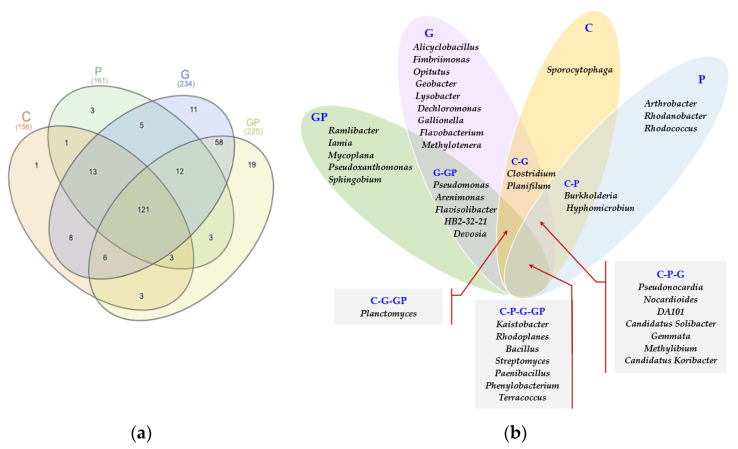
Effect of petrol (P) and cocksfoot (G) on (**a**) the number of unique and common bacterial genera, (**b**) unique and common genera of bacteria colonizing particular pots presented using the Veen diagram. C—non-polluted and non-sown soil, G—non-polluted soil sown with cocksfoot, P—non-sown soil polluted with petrol, and GP—soil polluted with petrol and sown with cocksfoot.

The Venn diagram (Figure 5a), depicting the number of all bacterial genera identified in particular pots, demonstrated that as many as 121 genera were common for all soil samples, regardless of soil cultivation and pollution. The highest number of specific bacterial genera was identified in the petrol-polluted soil sown with cocksfoot (19 genera), a slightly lower one in the soil sown with cocksfoot but non-polluted (11 genera), and the lowest one in the non-sown and non-polluted soil (1 genus) and in the non-sown but petrol-polluted soil (3 genera). After excluding bacterial genera with OTU lower than 1% of the assigned sequences, it can be undoubtedly concluded that *Kaistobacter*, *Rhodococcus, Bacillus*, *Streptomyces*, *Paenibacillus*, *Phenylobacterium*, and *Terracoccus* were the endogenous genera, hence common for oil soil types. In turn, *Arthrobacter*, *Rhodanobacter*, and *Rhodococcus* were found typical of the now-sown soil polluted with petrol, whereas *Ramlibacter*, *Iamia*, *Mycoplana*, *Pseudoxanthomonas*, and *Sphingobium* for the petrol-polluted soil sown with cocksfoot (Figure 5b).

**Figure 6:**
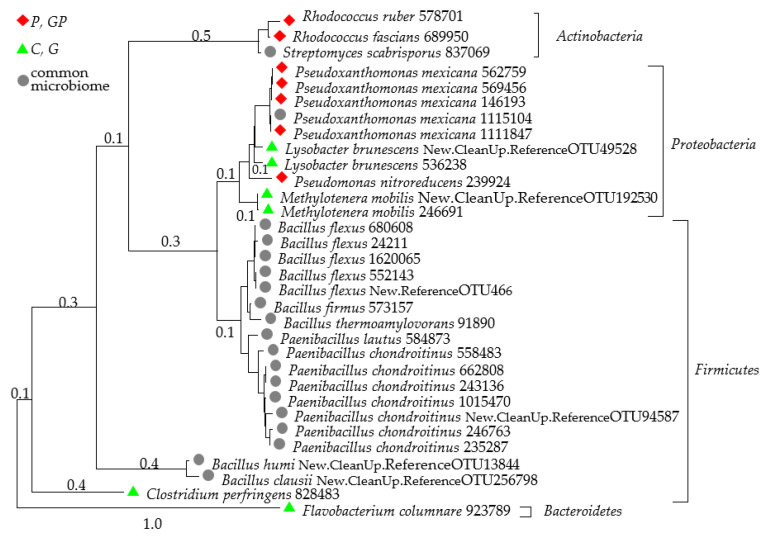
Phylogenetic tree of unique and common bacterial species. C—non-polluted and non-sown soil, G—non-polluted soil sown with cocksfoot, P—non-sown soil polluted with petrol, and GP—soil polluted with petrol and sown with cocksfoot.

Taking into account the types of bacterial genera with OTU higher than 1% of the assigned sequences, bacteria belonging to 10 genera were identified to the species (Figure 6). Although the *Proteobacteria* z bacteria were found to prevail, the highest number of sequences assigned to the species was identified for the phylum *Firmicutes* bacteria, which represented a common microbiome for all analyzed objects, both the non-polluted and the petrol-polluted ones.

**Figure 7:**
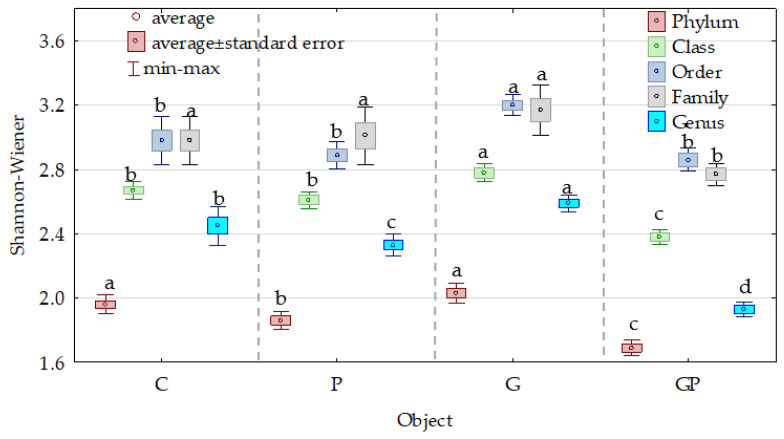
Effect of petrol (P) and cocksfoot (G) on the bacteria diversity, at particular taxonomic levels, estimated using the Shannon–Wiener index. C—non-polluted and non-sown soil, G—non-polluted soil sown with cocksfoot, P—non-sown soil polluted with petrol, and GP—soil polluted with petrol and sown with cocksfoot. Homogeneous groups denoted with letters (a–d) were calculated separately for each taxon.

Both, the Venn diagrams (Figure 5a,b) and values of the Shannon–Weaver index indicate (Figure 7) that cocksfoot had the most beneficial effect on the bacterial diversity at each taxonomic level.

### 2.2. Biochemical Properties of Soil

**Figure 8:**
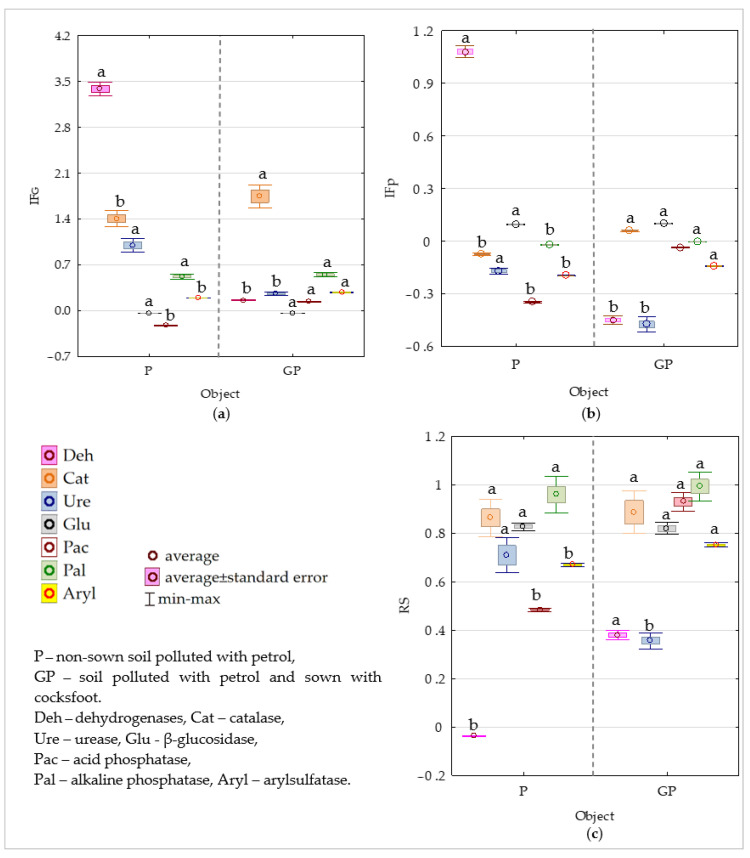
Indices of: (**a**) cocksfoot effect (IF_G_) on the activity of soil enzymes, (**b**) petrol effect (IF_P_) on the activity of soil enzymes, and (**c**) resistance (RS) of soil enzymes to the effects of petrol (P). Homogeneous groups denoted with letters (a,b) were calculated separately for each enzyme.

In the non-polluted soil (Table S2, Figure 8a) cocksfoot stimulated activities of dehydrogenases (Deh), catalase (Cat), urease (Ure), alkaline phosphatase (Pal), and arylsulfatase (Aryl), but inhibited activities of acid phosphatase (Pac), and β-glucosidase (Glu). In the petrol-polluted soil, it enhanced activities of most enzymes, but usually to a significantly lesser extent than in the petrol-polluted soil. The impact of petrol on the enzymatic activity was significantly lower compared to cocksfoot. In the non-sown soil, negative values of the petrol effect index were noted for all enzymes except for dehydrogenases and β-glucosidase, whereas in the cocksfoot-sown soil, the negative IF_P_ values were noted for Deh, Ure, Pac, and Aryl, and the positive ones for Cat and Glu (Figure 8b). The values of the resistance index (RS) showed that Pal was the most stable enzyme in the petrol-polluted soil that was sown and non-sown with cocksfoot, followed by Cat and Glue, and that Deh were the least stable (Figure 8c).

### 2.3. Degradation of Petrol

**Figure 9:**
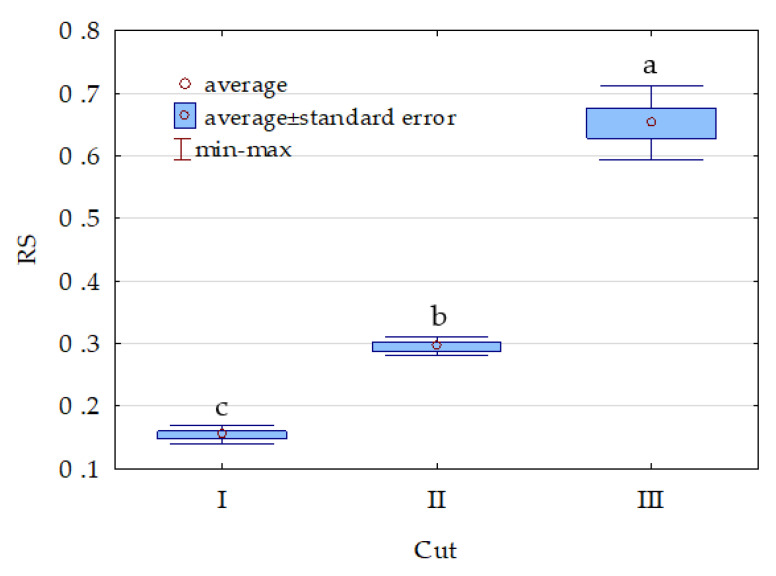
Index of resistance (RS) of cocksfoot to the effects of petrol. Homogeneous groups denoted with letters (a,b,c) were calculated separately for each of hydrocarbons.

Cocksfoot significantly accelerated degradation of the following hydrocarbons in soil: mineral oils (C_12_–C_35_), benzene (Ben), naphthalene (Nap), anthracene (Ant), chrysene (Chr), benzo[a]anthracene (BaA), benzo(a)pyrene (BaP), benzo[b]fluoranthene (BbF), and benzo(k)fluoranthene (BkF) (Table 1). The transformation of gasoline fractions (C_6_–C_12_), ethylbenzene (EtB), toluene (Tol), and xylene (Xyl) in the study period reached almost 100% and did not depend on soil cultivation type. In turn, the degradation of such hydrocarbons as Ant, Chr, BaA, BaP, BbF, BkF, and IP was much slower and, except for IP, was intensified by cocksfoot cultivation. Furthermore, cocksfoot itself was susceptible to the pollution with petrol; however, its resistance to the pollution increased with time. The resistance index (RS) of the first regrowth reached 0.154, that of the second regrowth reached 0.295, and that of the third regrowth reached 0.652 (Figure 9).

## 3. Discussion

### 3.1. Response of Microorganisms and Enzymes to Soil Pollution with Petrol

The soil microorganisms quite faithfully respond to ongoing changes in the soil; hence, they can be used as highly sensitive biosensors [30]. They influence the trends and magnitude of changes, leading to a specific homeostasis reached in ecosystems [68]. Environmental pollution can modify the microbiological succession and change its direction, debilitating ecosystem’s resistance [21,66]. In the present study, soil pollution with petrol significantly promoted the proliferation of all tested groups of microorganisms, as evidenced by positive IF_P_ values. In the soil non-sown with cocksfoot, petrol increased the count of bacteria from the *Proteobacteria* and *TM7* phyla, and reduced counts of those from *Actinobacteria* and *Chloroflexi* phyla. Furthermore, Galitskaya et al. [69] demonstrated that 12% soil pollution with petroleum for 120 days contributed to the succession of bacterial communities. The polluted soil was predominated by *Actinobacteria* (from 35% to 58%), *Proteobacteria* (from 25% to 30%), and *TM7* (from 15% to 35%).

In our experiment, the structure of communities has changed as well, i.e., the contribution of k strategist bacteria (slowly-growing ones) and r strategist fungi (rapidly growing ones) increased. The changes triggered by petrol in the structure of soil bacteria communities stem from the different resistance of individual species to its toxic effect [12,43,66,70] and from the diversified possibilities of utilizing its hydrocarbons as sources of carbon, hydrogen, and energy [71].

Our study indicated that, regardless of the stress triggered by soil pollution with petrol, the stability of the diversity of bacterial communities was highly reliably described by the autochthonous microbiome being common for the non-polluted and petrol-polluted soils, represented by *Kaistobacter*, *Rhodococcus*, *Bacillus*, *Streptomyces*, *Paenibacillus*, *Phenylobacterium*, and *Terracoccus*. It is the bacteria of these genera that are active in the degradation of petrol products, which can be effective in the bio-augmentation of polluted areas. Galitskaya et al. [69] demonstrated that the prevailing OTUs in the soil exposed to petrol belonged to the *Rhodococcus* and *Mycobacterium* genera, whereas according to de la Cueva et al. [66], the most abundant OTUs were these of: *Acinetobacter*, *Pedomicrobium*, *Halomonas*, *Rhizobium*, *Cryobacterium*, *Pseudomonas*, *Lysobacter*, *Thermomonas*, and *Stenotrophomonas*. Thus, even the soils strongly polluted with petroleum-based products reveal a potential for bio-remediation [58,72].

Because it is feasible to only cultivate a fraction of soil microbiota [15], it seems essential to determine the biological activity of soil by assaying its enzymatic activity [73,74]. The soil enzymes are perceived as reliable soil quality indicators [17,75,76]. They are used to construct models for the assessment of potential soil fertility [28,77,78,79,80]. Due to their ability to signalize changes triggered under the pressure of stress factors, they also perfectly mirror the effects of pollutants on soil [81,82,83]. Among the soil enzymes, dehydrogenases prove best in reflecting the rate of changes in the soil environment. They indicate levels of physiologically-active microorganisms in the soil, the oxidation rate of organic matter, and the availability of nutrients in the soil [71,76]. Therefore, in the present study, the petrol pollution of soil not-sown with cocksfoot caused an over 100% increase in the activity of dehydrogenases. Although more rarely used to determine pollution’s effect on the soil ecosystem’s stability, also such enzymes as catalase, β-glucosidase, urease, phosphatases, and arylsulfatase can provide valuable information on the metabolic capability of soil [73,74,83]. Their indicatory potential is due to the strong association of their activities with biogeochemical cycles of C, N, P, and S [71,75]. Our study has emphasized the significant effect of cocksfoot and petrol on C, N, and P metabolism in the soil, as evidenced by the changes in the population numbers and diversity of microorganisms as well as in activities of the soil enzymes tested. The present study proved alkaline phosphatase followed by catalase and β-glucosidase to be relatively the most stable enzymes, while dehydrogenases to be the least stable ones in the petrol-polluted soil.

### 3.2. The Role of Cocksfoot in Restoring the Biological Homeostasis of Petrol-Polluted Soil

The diversity of microorganisms and the activity of enzymes in the soil affect the functioning of ecosystems and, hence, the growth and health of plants [68]. For this reason, the petrol-induced disorders in the soil microbiome could deteriorate the physical properties of soil [35,36,37], leading to the poorer development of the root system and aerial parts of cocksfoot. Growth and development of the plants were hampered despite soil fertilization with nitrogen, phosphorus, potassium, and magnesium in doses meeting the cocksfoot demands for nutrients.

The present study proved that the negative response of cocksfoot to petrol diminished over time, which proves the progressing microbiological degradation of petrol hydrocarbons with time. Camacho-Montealegre et al. [42] demonstrated that, under favourable conditions, from 66% to 75% of hydrocarbons contained in petroleum products can be mineralized within 60 days depending on the species used for phytoremediation. In our study, cocksfoot proved effective in petrol degradation, and this effect was mainly due to its stimulating impact on soil microorganisms. The microbiological degradation of petroleum hydrocarbons in the soil is a natural process, owing to which organic pollutants can be mineralized by autochthonous microorganisms [41,84]. The cultivation of cocksfoot enhanced their activities as a result of the stimulating effect of root secretions on their development [42,60].

Petroleum hydrocarbons exhibit various susceptibilities to the attacks of microorganisms. The rate of their degradation decreases along with the elongation of the hydrocarbon chain [58,69,85]. Furthermore, in the present study, a higher degradation rate was observed for di-, tri-, and four-ring PAHs, followed by the five- and six-ring ones. According to Ite and Ibok [53], the effectiveness of biodegradability of oil components is as follows: n-alkanes > branched alkenes > low molecular weight n-alkyl aromatics > monoaromatics > cyclic alkanes > polycyclic aromatic hydrocarbons > asphaltene. Degradation of hydrocarbons in the soil is strongly associated with root secretions that ensure the rhizodegradation of organic pollutants [85,86,87].

Cocksfoot significantly intensified the degradation of all PAHs, except for IP, because plant roots improve the physical [35,36,37] and chemical [75] properties of soil, thereby creating more favourable conditions for the proliferation of microorganisms, including the hydrocarbon-degrading ones [87]. These abilities of plants are particularly important in the soils polluted with petroleum products [41,85], especially that plant roots create a specific habitat for rhizospheric microbial communities, which can be used for rhizoremediation [42,60]. The cultivation of cocksfoot on the petrol-polluted soil had an especially beneficial effect on the bacteria belonging to the phylum *Proteobacteria*, mainly those from the genus *Ramlibacter* and the class *β*-*Proteobacteria*, those from the genus *Pseudoxanthomonas* and the class *γ*-*Proteobacteria*, as well as those from *Mycoplana* and *Sphingobium* genera from the class *α*-*Proteobacteria.* Cocksfoot increased the bacterial diversity at each taxonomic level.

The results of our study demonstrate that petrol disturbed the stability of a soil ecosystem and confirm the hypothesis that cocksfoot is utile in restoring the biological homeostasis of soil contaminated with this pollutant. These findings suggest the need for continuous search, development, and implementation of bioremediation strategies for petrol-polluted soils.

## 4. Materials and Methods

### 4.1. Petrol

The study was conducted with petrol available at BP fuel stations [88] under a commercial name BP 98 unleaded petrol with Active technology. Apart from volatile hydrocarbons, it contains paraffins, naphtenes, olephins, and aromatic compounds. It is enriched with dirt-capturing substances forming a protective layer of the engine. Petrol characteristics is available at BP’s website [89].

### 4.2. Field Studies

The first stage of the study involved soil selection and evaluation of its properties (Table S3). Soils of arable lands with a little degree of anthropogenic transformations were in the focus of interest. Top quality soils with natural contents of elements and organic compounds were found in the north-eastern Poland. The soil to be used in the greenhouse experiment was collected from an arable field located near the city of Olsztyn (53.7167° N, 20.4167° E). This was Eutric Cambisol type soil with the following composition: 2.22% of the loam fraction, 22.85% of the dust fraction, and 74.93% of the sand fraction. Its samples were collected from the topsoil (0–20 cm). Its cation exchange capacity was 60.40 mmol (+) kg^−1^ d.m. soil, and it had pH_KCl_ 6.7. The analysed soil contained 9.3 g C_org_ kg^−1^ d.m. soil, 0.62 g N kg^−1^ d.m. soil, 93.68 mg available P kg^−1^ d.m. soil, 141.10 mg available K kg^−1^ d.m. soil, and 42 mg available Mg kg^−1^ d.m. soil. It had a very low content of PAHs, in mg kg^−1^ d.m. soil: gasoline fractions (C_6_–C_12_)—0.8; mineral oil (C_12_–C_35_)—6; benzene (Ben)—0.01; ethylbenzene (EtB)—0.01; toluene (Tol)—0.01; xylene (X)—0.03; naphthalene (Nap)—0.005; anthracene (Ant)—0.005; chrysene (Chr)—0.013; benzo[a]anthracene (BaA)—0.009; benzo(a)pyrene (BaP)—0.011; benzo[b]fluoranthene (BbF)—0.009; benzo(k)fluoranthene (BkF)—0.008; and indeno(1.2.3-cd)pyrene (IP)—0.006.

### 4.3. Greenhouse Experiment

Samples of soil (Eutric Cambisol) were transported to a greenhouse, thoroughly mixed, and sieved through a screen with a mesh diameter of 5 mm. The experiment was conducted in 7 dm^3^ Kick-Brauckman polyethylene pots, filled with 9 kg of soil. Four independent variables were tested in four replications, i.e., non-sown soil (C), non-sown soil polluted with petrol (P), soil sown with cocksfoot (G), and soil sown with cocksfoot and polluted with petrol (GP). Cocksfoot (*Dactylis glomerata*) was selected for this study as it easily adapts to unfavourable environmental conditions [90,91,92]. It is a fast-growing, loose-cluster species with a well-developed bundle root system, common to Europe, Asia, and North Africa. It is resistant to low temperatures, drought, and diseases [93,94]. It is also an energy grass, with biomass production reaching from 11 to 13 Mg d.m. per 1 ha [95]. The additional advantage of this grass species, speaking for its use in the phytoremediation of petrol-polluted soil, is its high resistance to the pollution with polycyclic aromatic hydrocarbons [96]. The same mineral fertilization was applied in each experimental series, in mg kg^−1^ d.m. soil: N—80, P—20, K—40, and Mg—10. Nitrogen was used in the form of urea, phosphorus in the form of potassium dihydrophosphate, while potassium as potassium dihydrophosphate and potassium chloride, and magnesium as magnesium sulfate heptahydrate. In P and GP pots, the soil was polluted with BP 98 unleaded petrol with Active technology in a dose of 7 cm^3^ kg^−1^ d.m. soil. The mineral fertilizer and petrol were applied in a single dose before pot filling with the soil, after their separate mixing with each soil portion (9 kg) intended for one pot. Afterward, soil moisture content was brought to 60% of the water capillary capacity using distilled water. One week after the experiment had been established, 24 seeds of cocksfoot (*Dactylis glomerata*) were sown in pots G and GP. The experiment was continued for 105 days. On day 45, 75, and 105, cocksfoot was cut, and its dry matter yield was determined. The soil moisture content was kept stable throughout the experiment at 60% of the water capillary capacity. During the last cut, soil samples were collected for microbiological and biochemical analyses. The samples were sifted through a screen with mesh diameter of 2 mm.

### 4.4. Microbiological Analyses

#### 4.4.1. Determination of Population Numbers of Microorganisms

A serial dilution method was used to determine population numbers of organotrophic bacteria, actinobacteria, and fungi in the soil samples. To this end, serial dilutions from 10^−2^ to 10^−6^ were prepared from the samples of non-polluted and petrol-polluted soil. One cm^3^ of a respective soil dilution (10^−5^ and 10^−6^ for organotrophic bacteria and actinobacteria, and 10^−3^ and 10^−4^ for fungi) was measured onto Petri dishes. Then, 18 cm^3^ of a selective medium (organotrophic bacteria—Bunt and Rovira medium, actinomycetes—Parkinson medium, and fungi—Martin medium) were added onto the dishes. The medium’s composition was presented in our earlier work [97]. Incubation was performed at a temperature of 28 °C for 10 days. The colony forming units (cfu) of microorganisms were counted every 24 h for 10 consecutive days with a colony counter. The microbiological analyses were performed in 4 replications.

#### 4.4.2. DNA Isolation

Genomic DNA was isolated from the samples of non-polluted and petrol-polluted soil using a Genomic Minix X Soil kit, following producer’s guidelines. To check isolated DNA quality and its bacterial origin, a Real-Time PCR was conducted in the Mx3000P thermocycler (Stratagene) using the SYBR Green dye as a fluorochrome. Two universal primers were used, i.e., 1055F and 1392R.

#### 4.4.3. 16S rRNA Gene Amplicon Sequencing

Bacterial communities colonizing the non-polluted and petrol-polluted soils were analysed by sequencing the hypervariable region V3–V4 of the 16S rRNA gene. The mass sequencing of 16S rRNA amplicons were performed by Genomed SA, Poland, following the Illumina protocol. A fragment of the 16S rRNA gene was amplified with PCR primers recommended for the Illumina technique, i.e., 341F and 785R. DNA was sequenced on the Illumina MiSeq apparatus in the paired-end 2 × 250 protocol, using the Miseq Reagent Kit v2 (Illumina, San Diego, CA, USA).

#### 4.4.4. Bioinformatic Analysis

The quality of the obtained sequences was controlled, and incomplete or chimeric ones were rejected. The identified sequences were grouped using the uclust algorithm. The taxonomic identification was performed with the QIIME package using a database of reference sequences GreenGenes v13_8. The sequencing data were deposited in the GenBank NCBI [98] under accession numbers of MW266821-MW266860; MW380430-MW380501; MW579372-MW579410; MW686950-MW687007.

#### 4.4.5. Biochemical, Chemical, and Physicochemical Analyses of Soil

Activities of the following soil enzymes were determined in the study: dehydrogenases (Deh), catalase (Cat), urease (Ure), alkaline phosphatase (Pal), acid phosphatase (Pac), arylsulfatase (Aryl), and β-glucosidase (Glu). All determinations were carried out with standard methods described in our previous works [97] in three replications, under controlled conditions The enzymatic activity was expressed in product units per 1 kg d.m. soil per 1 h. The activity of Deh was expressed in μmol TFF (triphenyl formazane), that of Cat in mol O_2_, that of Ure in mmol N-NH_4_^+^, and these of Pal, Pac, Aryl, and Glu in mmol PN (4-nitrophenol).

In addition, the soil samples were determined for the contents of: gasoline fractions (C_6_–C_12_), mineral oils (C_12_–C_35_), volatile aromatic hydrocarbons (BETX), naphthalene (Nap), anthracene (Ant), chrysene (Chr), benzo(a)antracene (BaA), benzo(a)pyrene (BaP), benzo(b)fluoranthene (BbF), benzo(k)fluoranthene (BkF), and indeno(123-cd)pyrene (IP), following procedures provided in the following standards: EN ISO 18287 [99], EN ISO 16703 [100], and EN ISO 22155 [101]. The above determinations were conducted using an Agilent 7890A-5975C mas spectrometer equipped in EI/CI ion source.

The soils samples were also analysed for their granulometric composition; pH value; contents of total nitrogen, organic carbon, available P, K, and Mg; exchangeable cations Ca^2+^, Mg^2+^, K^+^, and Na^+^, and ion exchange capacity. All determinations were carried out with standard methods described in our previous work [70].

#### 4.4.6. Calculations and Statistical Analysis

The following indices were determined to establish cocksfoot and petrol effects on soil condition:s

(1) colony development index (CD) of organotrophic bacteria, actinobacteria, and fungi according to Leij et al. [102]:

CD = [N1/1 + N2/2 + N3/3….. N10/10] · 100,(1)

where: N1, N2, N3,...N10—sum of the quotients of colony numbers of microorganisms identified in particular days of the study (1, 2, 3,...10) and the sum of all colonies in the entire study period;

(2) ecophysiological diversity index (EP) of organotrophic bacteria, actinobacteria, and fungi according to Leij et al. [102]:

EP = −Σ(π·log_10_ π), (2)

where: pi—the quotient of the number of colonies of microorganisms from particular days of the study and the sum of all colonies from the entire study period;

(3) index of cocksfoot effect (IF_G_) on soil microorganisms and enzymes:

(3) $${\mathrm{IF}}_{G}=\frac{A_{G}}{A_{0}}-1,$$

where: A_G_—the number of microorganisms or the activity of enzymes in the soil sown with cocksfoot, A_0_—the number of microorganisms or the activity of enzymes in the non-sown soil;

(4) index of petrol effect (IF_P_) on soil microorganisms and enzymes:

(4) $${\mathrm{IF}}_{P}=\frac{A_{P}}{A_{0}}-1,$$

where: A_p_—the number of microorganisms or the activity of enzymes in the soil polluted with petrol, A_0_—the number of microorganisms or the activity of enzymes in the non-polluted soil;

(5) resistance (RS) of microorganisms, enzymes, and cocksfoot to soil pollution with petrol [103]:

(5) $$\mathrm{RS}\;=1-\frac{2\;│D_{0}│}{C_{0}+│D_{0}│},$$

where: D_0_ = C_0_—P_0_, C_0_—parameter value determined for the control soil, P_0_—parameter value determined for the petrol-polluted soil;

(6) index of hydrocarbon degradation (ID_H_):

(6) $${\mathrm{ID}}_{H}=100-\frac{A_{H}\cdot 100\%}{A_{H0}},$$

where A_H0_—the content of hydrocarbons in the petrol-polluted soil on the day of experiment establishment, A_H_—the content of hydrocarbons on the day of experiment termination.

(7) Shannon–Weaver index (H) determining bacterial diversity:

H = −Σp_i_(lnp_i_), (7)

where: p is the ratio of OTU numbers of one representative of the tested taxon to the total OTU number of the entire taxon.

The normality of data distribution was verified with the Kruskal–Wallis and Shapiro–Wilk tests. The post-hoc Duncan test was used for further analyses. One-way significance tests (Table S2) were performed using the analysis of variance (ANOVA). Statistical calculations were performed with the Statistica 13.3 package [104]. The results of the metagenomic analysis were elaborated and interpreted using: STAMP 2.1.3. software [105], RStudio v1.2.5033 software [106], R v3.6.2 system [107], gplots libraries [108], and cluster analysis [109]. The STAMP 2.1.3. software was used to present significant differences between sequence proportions for the phylum in the analysed soil samples, at a confidence interval of 95%. The diversity of bacteria at the genus level was presented using the heatmap with a dendrogram of their similarities, generated using RStudio v1.2.5033 software [106], R v3.6.2 system [107] and gplots library [108] as well as a Venn diagram plotted using InteractiVenn [109].

## 5. Conclusions

The microbiological and biochemical homeostasis was disturbed in the soil polluted with petrol. The pollution resulted in increased counts of bacteria and fungi as well as enhanced enzymatic activity. It also caused the increased contribution of k strategist bacteria and r strategist fungi in the structure of respective communities. Soil sowing with cocksfoot increased the genetic diversity of bacteria and accelerated the degradation of petrol hydrocarbons, which predisposes this plant for phytoremediation purposes. *Proteobacteria*, *Actinobacteria*, and *Acidobacteria* were the prevailing phyla in the soil tested. Its pollution with petrol and sowing with cocksfoot increased the abundance of *Proteobacteria* and decreased the abundance of *Actinobacteria* and *Acidobacteria.* The ones least susceptible to the soil pollution with petrol and cocksfoot cultivation were the bacteria of the following genera: *Kaistobacter*, *Rhodoplanes*, *Bacillus*, *Streptomyces*, *Paenibacillus*, *Phenylobacterium*, and *Terracoccus*.

## Figures and Tables

**Table 1:** Effect of cocksfoot on degradation of petrol hydrocarbons in the soil, in %.

**Object**	**C_6_–C_12_**	**C_12_–C_35_**	**Ben**	**EtB**	**Tol**	**Xyl**	**Nap**
P	99.53 ± 1.06 ^a^	71.41 ± 0.70 ^b^	95.81 ± 0.09 ^b^	99.83 ± 0.09 ^a^	99.86 ± 0.15 ^a^	99.87 ± 0.10 ^a^	90.63 ± 0.09 ^b^
GP	99.76 ± 0.90 ^a^	76.56 ± 0.69 ^a^	98.11 ± 0.19 ^a^	99.95 ± 0.13 ^a^	99.96 ± 0.20 ^a^	99.96 ± 0.13 ^a^	99.84 ± 0.09 ^a^
**Object**	**Ant**	**Chr**	**BaA**	**BaP**	**BbF**	**BkF**	**IP**
P	59.09 ± 0.14 ^b^	18.33 ± 0.10 ^b^	40.91 ± 0.09 ^b^	35.00 ± 0.07 ^b^	16.92 ± 0.04 ^b^	13.33 ± 0.07 ^b^	40.00 ± 0.08 ^a^
GP	84.85 ± 0.11^a^	41.67 ± 0.09 ^a^	54.55 ± 0.11 ^a^	50.00 ± 0.10 ^a^	30.77 ± 0.06 ^a^	33.33 ± 0.07 ^a^	40.00 ± 0.08 ^a^

C_6_–C_12_—gasoline fractions; C_12_–C_35_—mineral oil; Ben—benzene; EtB—ethylbenzene; Tol—toluene; X—xylene; Nap—naphthalene; Ant—anthracene; Chr—chrysene; BaA—benzo[a]anthracene; BaP—benzo(a)pyrene; BbF—benzo[b]fluoranthene; BkF—benzo(k)fluoranthene; IP—indeno(1.2.3-cd)pyrene. P—non-sown soil polluted with petrol. GP—soil polluted with petrol and sown with cocksfoot. Homogeneous groups denoted with letters (a,b) were calculated separately for each of hydrocarbons.

## Data Availability

The data presented in this study are available on request from the corresponding author.

## Supplementary Materials

The following are available online. Table S1: Effect of petroleum (P) and cocksfoot (G) on the count of organotrophic bacteria (Org), actinobacteria (Act), and fungi (Fun) in the soil, 10^n^ cfu kg^−1^ soil d.m. n—for Org and Act = 9, and for Fun = 7, Table S2: Effect of petroleum (P) and cocksfoot (G) on the activity of enzymes in 1 kg of soil d.m. within 1 h, Table S3: Main properties of soil used in the study, Figure S1: Effect of petroleum on the yield of cocksfoot, d.m. in g per pot.

## Sample Availability

Not available.
