# Supplementary material for: Microbiological Study in Petrol-Spiked Soil
Source: Molecules. 2021 May 1;26(9):2664. doi: 10.3390/molecules26092664 (PMC8125633; doi:10.3390/molecules26092664)
Supplement: Supplementary file 1 [file molecules-26-02664-s001.zip › molecules-1197604-supplementary.pdf]

molecules

Supplementary materials

Microbiological Study in Petrol-Spiked Soil

Agata Borowik, Jadwiga Wyszkowska* and Jan Kucharski

[1] Department of Soil Science and Microbiology, University of Warmia and Mazury in Olsztyn, Poland; agata.borowik@uwm.edu.pl (A.B.); jadwiga.wyszkowska@uwm.edu.pl (J.W.); jan.kucharski@uwm.edu.pl (J.K.)

* Correspondence: jadwiga.wyszkowska@uwm.edu.pl

Table S1. Effect of petrol (P) and cocksfoot (G) on the count of organotrophic bacteria (Org), actinobacteria (Act), and fungi (Fun) in the soil, 10^n cfu kg^{-1} soil d.m. n – for Org and Act = 9, and for Fun = 7.

Object	Org · 10^9	Act · 10^9	Fun · 10^7
C	12.361 ± 0.589 [d]	11.658 ± 1.281 [d]	3.216 ± 0.353 [c]
P	18.362 ± 0.874 [b]	20.865 ± 0.993 [b]	4.956 ± 0.236 [b]
G	15.394 ± 0.733 [c]	13.543 ± 0.645 [c]	5.237 ± 0.249 [b]
GP	25.381 ± 1.208 [a]	28.600 ± 1.362 [a]	7.159 ± 0.341 [a]

Homogeneous groups denoted with letters (a-d) were calculated separately for each of the microorganisms. C - non-polluted and non-sown soil, G - non-polluted soil sown with cocksfoot, P - non-sown soil polluted with petrol, GP - soil polluted with petrol and sown with cocksfoot.

Table S2. Effect of petrol (P) and cocksfoot (G) on the activity of enzymes in 1 kg of soil d.m. within 1 h.

Object	Deh	Cat	Ure	Glu	Pac	Pal	Aryl
	μmol TFF	mol O_2	mmol $N\text{-}NH_4$	mmol PNP			
C	0.666 ± 0.032 [d]	0.098 ± 0.005 [c]	0.364 ± 0.009 [b]	0.277 ± 0.008 [b]	1.201 ± 0.057 [a]	0.160 ± 0.008 [b]	0.102 ± 0.005 [b]
P	1.384 ± 0.048 [c]	0.091 ± 0.006 [c]	0.302 ± 0.010 [c]	0.303 ± 0.008 [a]	0.785 ± 0.037 [c]	0.157 ± 0.007 [b]	0.082 ± 0.004 [c]
G	2.920 ± 0.119 [a]	0.235 ± 0.010 [b]	0.727 ± 0.020 [a]	0.265 ± 0.008 [b]	0.930 ± 0.044 [b]	0.244 ± 0.012 [a]	0.122 ± 0.003 [a]
GP	1.609 ± 0.051 [b]	0.249 ± 0.010 [a]	0.382 ± 0.016 [b]	0.291 ± 0.011 [a]	0.897 ± 0.043 [b]	0.243 ± 0.012 [a]	0.105 ± 0.005 [b]

Homogeneous groups denoted with letters (a-d) were calculated separately for each enzyme. C - non-polluted and non-sown soil, P - non-sown soil polluted with petrol, G - non-polluted soil sown with cocksfoot, GP - soil polluted with petrol and sown with cocksfoot, Deh - dehydrogenases, Cat – catalase, Ure – urease, Glu - β-glucosidase, Pac – acid phosphatase, Pal – alkaline phosphatase, Aryl – arylsulfatase

Table S3. Main properties of soil used in the study

Granulometric subgroup	Particle diameter, mm			pH_{KCl}	N_{total}	C_{org}
	2.0-0.050	0.05-0.002	<0.002			
	%				g kg^{-1}	
sandy loam	74.93 ± 0.19	22.85 ± 0.11	2.22 ± 0.01	6.7 ± 0.01	0.62 ± 0.00	9.30 ± 0.02

available forms mg kg^{-1} d.m. of soil			exchangeable cations g kg^{-1} d.m. of soil			
P	K	Mg	K_e	Ca_e	Na_e	Mg_w
93.68 ± 0.17	141.10 ± 0.42	42.00 ± 0.15	156.00 ± 0.40	623.50 ± 0.96	40.00 ± 0.10	59.50 ± 0.15

N_{total} – total nitrogen, C_{org} - total organic carbon, P - available phosphorus, K - available potassium, Mg - available magnesium, K_e – exchangeable potassium, Ca_e – exchangeable calcium Na_e – exchangeable sodium, Mg_e – exchangeable magnesium

Table S4. One-way significance tests carried out using the analysis of variance (ANOVA).

Object/Index		SS	MS	F	P
Numbers of microorganisms	Organotrophic bacteria	372.547	124.182	159.829	0.000
	Actinobacteria	715.509	238.503	194.834	0.000
	Fungi	31.286	10.429	116.256	0.000
RS	Organotrophic bacteria	0.035	0.035	21.261	0.004
	Actinobacteria	0.058	0.058	2021.224	0.000
	Fungi	0.054	0.054	16.243	0.007
IF_P	Organotrophic bacteria	0.054	0.054	38.674	0.001
	Actinobacteria	0.207	0.207	130.086	0.000
	Fungi	0.061	0.061	53.726	0.000
IF_G	Organotrophic bacteria	0.038	0.038	9.429	0.022
	Actinobacteria	0.087	0.087	23.156	0.003
	Fungi	0.070	0.070	14.545	0.009
Colony development index (CD)	Organotrophic bacteria	167.140	55.710	22.440	0.000
	Actinobacteria	107.262	35.754	14.564	0.000
	Fungi	90.210	30.070	3.947	0.036
Ecophysiological diversity index (EP)	Organotrophic bacteria	0.054	0.018	11.303	0.001
	Actinobacteria	0.009	0.003	1.121	0.379
	Fungi	0.262	0.087	23.515	0.000
Shannon-Weiner's diversity index	Phylum	0.261	0.087	30.510	0.000
	Class	0.342	0.114	34.630	0.000
	Order	0.284	0.095	11.590	0.001
	Family	0.324	0.108	6.254	0.008
	Genus	0.968	0.323	63.520	0.000
Enzymes	Dehydrogenases	10.614	3.538	702.445	0.000
	Catalase	0.088	0.029	449.698	0.000
	Urease	0.442	0.147	709.610	0.000
	Acid phosphatase	0.374	0.125	89.590	0.000
	Alkaline phosphatase	0.029	0.010	100.760	0.000
	Arylsulfatase	0.003	0.001	61.862	0.000
	β-glucosidase	0.003	0.001	13.760	0.000
RS	Dehydrogenases	0.349	0.349	2111.693	0.000
	Catalase	0.001	0.001	0.143	0.718
	Urease	0.251	0.251	72.622	0.000
	Acid phosphatase	0.396	0.396	619.531	0.000
	Alkaline phosphatase	0.002	0.002	0.602	0.467
	Arylsulfatase	0.013	0.013	297.800	0.000
	β-glucosidase	0.000	0.000	0.240	0.640
IF_P	Dehydrogenases	4.676	4.676	6356.273	0.000
	Catalase	0.035	0.035	872.472	0.000
	Urease	0.186	0.186	205.834	0.000
	Acid phosphatase	0.193	0.193	13852.870	0.000
	Alkaline phosphatase	0.001	0.001	626.594	0.000
	Arylsulfatase	0.006	0.006	2628.920	0.000
	β-glucosidase	0.000	0.000	5.560	0.056
IF_G	Dehydrogenases	20.814	20.814	4159.505	0.000
	Catalase	0.238	0.238	9.342	0.022
	Urease	1.083	1.083	173.102	0.000
	Acid phosphatase	0.271	0.271	13649.580	0.000
	Alkaline phosphatase	0.001	0.001	1.261	0.304
	Arylsulfatase	0.013	0.013	2571.990	0.000
	β-glucosidase	0.000	0.000	33.080	0.001
PAH`s	C_6-C_{12}	0.110	0.110	0.110	0.752
	C_{12}-C_{35}	53.050	53.050	109.090	0.000
	Ben	10.580	10.580	456.000	0.000
	EtB	0.030	0.030	2.000	0.177
	Tol	0.020	0.020	1.000	0.452
	Xyl	0.020	0.020	1.000	0.318
	Nap	169.650	169.650	20298.000	0.000
	Ant	1327.160	1327.160	84550.000	0.000
	Chr	1089.511	1089.511	132790.200	0.000
	BaA	372.100	372.100	37187.000	0.000
	BaP	450.000	450.000	56257.000	0.000
	BbF	383.645	383.645	145901.000	0.000
	BkF	800.000	800.000	168672.500	0.000
	IP	0.000	0.000	0.000	1.000
The yield of grasses	Cut I	1105.346	1105.346	254.230	0.000
	Cut II	176.307	176.307	636.404	0.000
	Cut III	12.251	12.251	37.525	0.001

F – F-test; P – level of probabilisty.

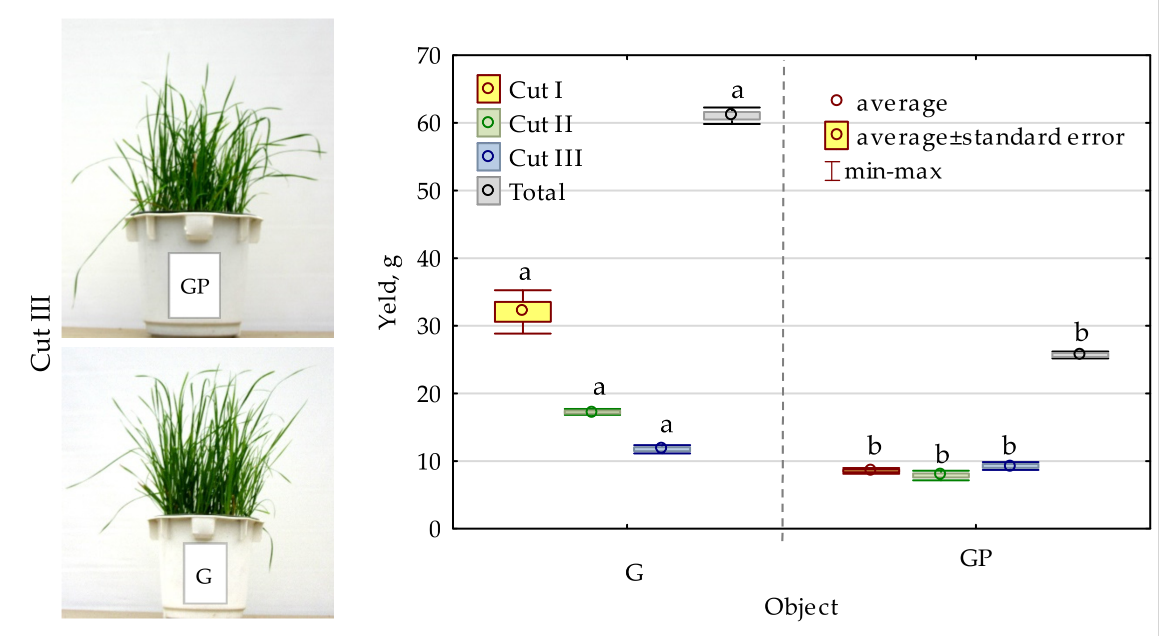

Figure S1. Effect of petrol on the yield of cocksfoot, d.m. in g per pot. G - non-polluted soil sown with cocksfoot, GP - soil polluted with petrol and sown with cocksfoot.
